# Self-report versus electronic medical record recorded healthcare utilisation in older community-dwelling adults: Comparison of two prospective cohort studies

**DOI:** 10.1371/journal.pone.0206201

**Published:** 2018-10-26

**Authors:** Emma Wallace, Frank Moriarty, Christine McGarrigle, Susan M. Smith, Rose-Anne Kenny, Tom Fahey

**Affiliations:** 1 HRB Centre for Primary Care Research, Royal College of Surgeons in Ireland, Dublin, Ireland; 2 The Irish Longitudinal Study of Ageing, Lincoln Gate, Trinity College Dublin, Dublin, Ireland; Brown University Warren Alpert Medical School, UNITED STATES

## Abstract

**Introduction:**

Self-reported measures of healthcare utilisation are often used in longitudinal cohort studies involving older community-dwelling people. The aim of this study is to compare healthcare utilisation rates using patient self-report and manual extraction from the general practice (GP) electronic medical record (EMR).

**Methods:**

Study population: Two prospective cohort studies (n = 806 and n = 1,377, aged ≥70 years) conducted in the Republic of Ireland were compared. Study outcomes: GP, outpatient department (OPD) and emergency department (ED) visits over a one-year period. *Statistical analysis*: Descriptive statistics of the two cohorts are presented. A negative binomial regression was performed and results are presented as incidence rate ratios (IRR) with 95% confidence intervals (CI). For the outcome of any ED visit, linear regression was performed, yielding risk ratios (RR) with 95% CI.

**Results:**

The annual rates of GP, OPD and ED visits were 6.30 (SD 4.63), 2.11 (SD 2.46) and 0.26 (SD 0.62) respectively in GP EMR cohort, compared to 5.65 (SD 8.06), 2.09 (SD 5.83) and 0.32 (SD 0.84) in the self-report cohort. In univariate regression analysis comparing healthcare utilisation, the self-report cohort reported a lower frequency of GP visits (unadjusted IRR 0.90 (95% CI 0.84, 0.96), p = 0.02)), a greater frequency of ED visits (1.20 (0.98, 1.49), p = 0.083)), and no difference in OPD visits (unadjusted IRR 0.99 (95% CI 0.86, 1.13), p = 0.845)). In multivariate analysis, adjusted for relevant confounders, there was no difference in GP visits (adjusted IRR 0.99 (95% CI 0.92, 1.06), p = 0.684)) or OPD visits (adjusted IRR 1.09 (0.95, 1.25), p = 0.23)) between the two cohorts. However, the self-report cohort reported 37% more ED visits (adjusted IRR 1.37 (1.10, 1.71), p = 0.005)) and were more likely to report any ED visit (adjusted RR 1.23 (95% CI 1.02, 1.48), p = 0.028)).

**Conclusions:**

This study demonstrates that reported rates of GP and OPD visits were similar but there were differences in reported ED visits, with significantly higher self-reported visits. This may be due to ED visits not being notified to the GP and contextual issues such as transfer of healthcare utilisation data between sectors may vary in different healthcare systems.

## Introduction

Self-reported measures of healthcare utilisation are often used in longitudinal cohort studies involving older community dwelling people, usually via surveys or interview.[1–4] Self-reported healthcare use is valid across socioeconomic groups, and research indicates that younger people, those with higher education and healthier individuals may recall more accurately.[3, 5] Self-report measures also have the advantage of being less time consuming to collect, compared to extracting data directly from the general practice (GP) electronic medical record (EMR).

For older people, self-report is most accurate for salient events such as emergency admission and emergency department (ED) visits.[6–9] However, there are concerns that other types of healthcare utilisation are underestimated using this approach, including GP visits and outpatient department (OPD) visits.[6, 7] Reduced concordance between self-report and EMR data is more likely with longer recall periods and with increased frequency of events, where patients are more likely to under-report their healthcare use.[6, 9–12] This has implications if relying solely on self-report data to extrapolate for broader healthcare planning and healthcare expenditure purposes. Previous studies have had some methodological limitations (cross sectional study design) and there is a need to examine this issue in prospective cohort studies involving older community-dwelling people, allowing adjustment for confounders over time.[7, 10]

The aim of this study is to examine longitudinally patient self-reported and GP EMR recorded healthcare utilisation (GP, OPD and ED visits), in older community-dwelling people. Healthcare utilisation rates in two established longitudinal cohort studies are compared, one using patient self-report and the other manual extraction of healthcare utilisation data from the GP EMR.

## Methods

### Design, setting and study population

The Strengthening The Reporting of Observational Studies in Epidemiology (STROBE) guidelines were adhered to in the conduct and reporting of this study.[13] Two prospective cohort studies conducted in the Republic of Ireland, one using self-report measures of healthcare utilisation and the other using manual extraction of healthcare utilisation from the GP EMR were compared. To ensure the populations examined are similar, community-dwelling people aged ≥70 years were included in the analysis. The two cohort studies are described in more detail below.

#### Centre for Primary Care research (CPCR) cohort (n = 904)

This is a two-year prospective cohort study of 904 older patients (aged ≥70 years) recruited from 15 general practices in the Leinster region of the Republic of Ireland (2010–2012). At baseline in 2010, a proportionate stratified random sampling approach was used to recruit patients for study participation. There were a total of 4,573 patients aged ≥70 years across the 15 practices, of whom a proportionate random sample were selected to participate (n = 1,764). A total of 1,487 patients remained eligible following application of exclusion criteria and a total of 904 (response rate = 61%) took part in the study at baseline.[14] A more detailed explanation of the methods has been published previously.[14, 15]

Study inclusion criteria were: i) aged ≥70 years on 1st January 2010; ii) in receipt of a valid general medical services (GMS) scheme card (this entitled holders to free medical care, including GP and hospital care, and a range of other benefits) and, iii) taking at least one regular medication. The GMS scheme is a form of public health cover in Ireland where eligibility based on household income and age, and approximately 90% of those aged 70 years or older are eligible. As this study required participants to complete a postal questionnaire and a telephone interview the following exclusion criteria were applied: i) Receiving palliative care; ii) Cognitive impairment at the level that would impact their ability to complete the outcome measure (defined as Mini Mental State Examination (MMSE) ≤20); iii) Significant hearing/speech/visual impairment; iv) Currently experiencing a psychotic episode; v) Hospitalised long-term, in a nursing home, homeless or in sheltered accommodation; and, vi) Recent bereavement (within four weeks). Each participant’s GP applied the exclusion criteria and determined eligibility for participation at baseline in 2010 and again at follow-up in 2012. Ethical approval for this study was granted by the Royal College of Surgeons in Ireland (RCSI) Human Research Ethics committee and all participants gave informed consent prior to participating.[14, 15]

**Fig 1:**
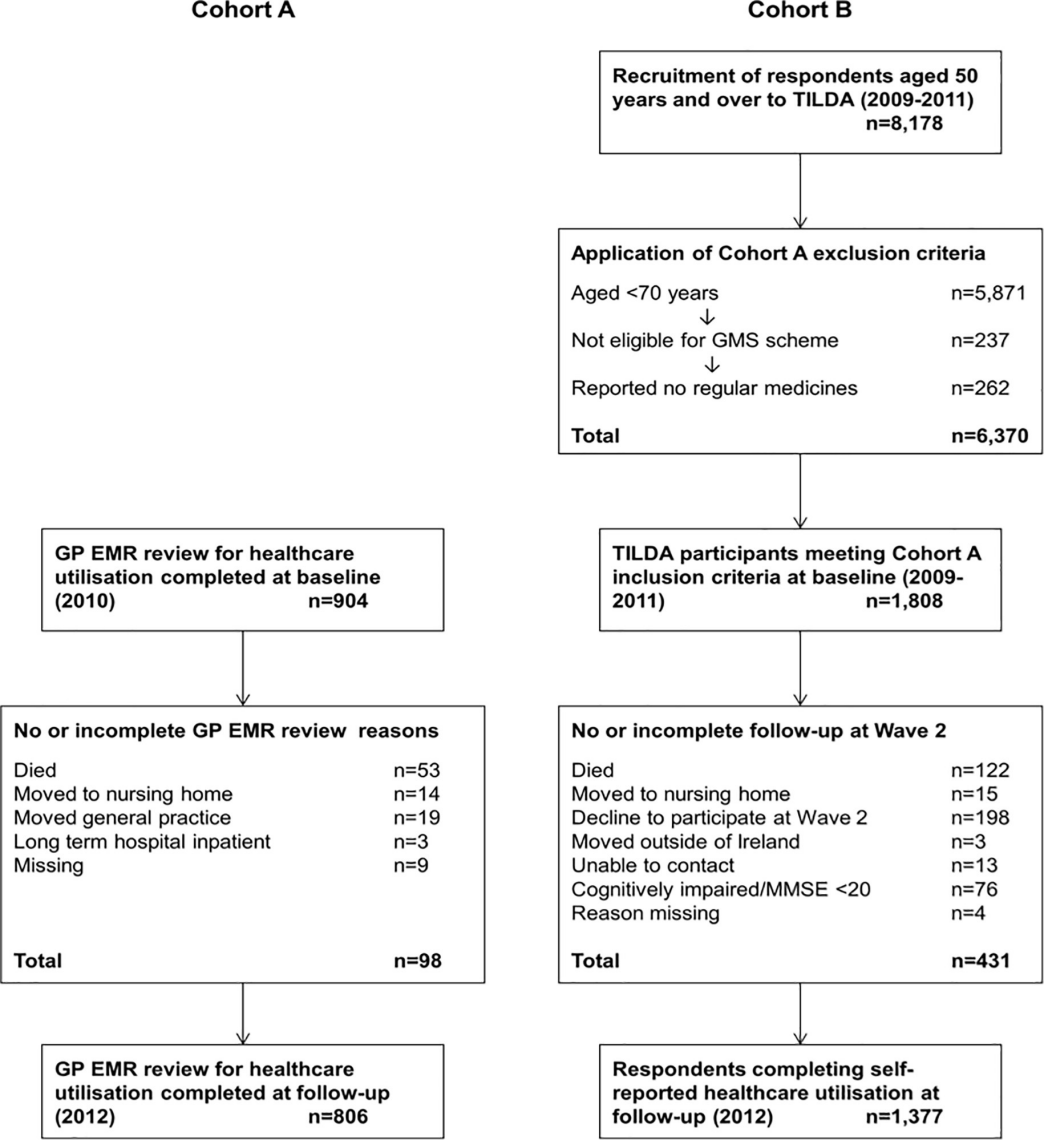
**Flow of participants through the CPCR cohort A (GP electronic medical record (EMR) review, 2010–2012) and TILDA cohort B (self-reported healthcare utilisation, 2009–2012)**.

A total of 859 (95%) of study participants had their GP EMR reviewed for healthcare utilisation at two year follow up. Of these, 53 had died resulting in 806 participants included in the present study. See Fig 1 for a flow diagram of study participants during the study and reasons for losses to follow up.

#### The Irish Longitudinal Study on Ageing (TILDA) cohort (n = 1,808)

The Irish Longitudinal Study on Ageing (TILDA) is a nationally representative cohort study of community-dwelling people aged ≥50 years in Ireland.[16] Between October 2009 and February 2011, a stratified clustered sample of representative individuals identified from a geodirectory of residential addresses using the RANSAM method were recruited. A total of 8,175 respondents aged 50 years and over from 6,282 households (response rate 62%) underwent an extensive face-to-face interview and were invited to a health assessment either at a dedicated centre or in their home. Follow-up was scheduled to occur every two years to chart the health, economic and social circumstances of individuals growing older in Ireland. Ethical approval for TILDA was granted by the Faculty of Health Sciences Research Ethics Committee at Trinity College Dublin and all respondents gave informed consent prior to participating.

As TILDA had broader inclusion criteria compared to the CPCR cohort, a subsample of respondents who would have been eligible for the CPCR cohort were included in this analysis. A total of 2,307 TILDA respondents were aged ≥70 years at baseline, 2,070 of whom (90%) were eligible for the GMS scheme. Those with a GP visit card were not included. At least one regular medication was reported by 1,808 of these respondents (87%). Follow-up at Wave 2 was completed by 1,377 (76%) of these respondents between February 2012 and January 2013, where a further interview was conducted. (See Fig 1).

### Outcomes and outcome measurements

The outcomes are rates of GP, OPD, and ED visits during the second 12 months of study follow-up, and any ED visit in this time period. The measurement of healthcare utilisation differed between the CPCR cohort and TILDA.

#### CPCR cohort–EMR-extraction (n = 904)

Healthcare utilisation was examined by measuring ED visits, OPD visits and GP visits. A detailed review of and data extraction from each participant’s electronic GP EMR was undertaken by researchers at baseline (2010) and again at follow-up (2012). For this two-year period the number of and date(s) of ED visits were recorded. These data was extracted from the correspondence section of the EMR and consultation notes were also reviewed to include any additional ED attendances. The number of OPD visits and speciality attended was recorded. The number of GP visits was recorded by manually counting patient visits recorded in the EMR. Of note, out of hours GP visits were not included and GP practice nurse visits were recorded separately.

In addition, patient demographic and clinical characteristics were recorded by EMR review (date of birth, gender, comorbidity), patient questionnaire (socioeconomic demographics) and by linkage to the national pharmacy claims database (number of drugs, medication possession ratio).[14, 15] The annual rate of ED visits during the second year of follow-up was determined by summing the number of ED visits (that did not result in an emergency hospital admission).

#### TILDA cohort–self-report (n = 1,808)

Healthcare utilisation was examined in TILDA by asking respondents during face-to-face interview to report the number of visits in the previous 12 months to various types of healthcare provider as a patient. This included GP visits, OPD visits and ED visits (see Box 1). There is no specific question regarding GP practice nurse visits in the TILDA interview.

**Box 1 Healthcare utilisation questions asked during baseline and 2 year follow-up TILDA interviews**

- In the last 12 months, about how often did you visit your GP?
- In the last 12 months, how many times did you visit a hospital Emergency Department (sometimes called A&E or Accident and Emergency) as a patient?
- In the last 12 months, about how many visits did you make to a hospital as an out-patient? (Include all types of consultations, tests, operations, procedures or treatments)
- In the last 12 months, on how many occasions were you admitted to hospital overnight?

Demographic characteristics gathered during interview included age, gender, social class, and level of education. During interview, respondents were also asked to show the medications that they take on a regular basis allowing the number of regular drugs to be determined.

### Exposure variable and covariates

The exposure variable of interest was whether healthcare utilisation data were self-reported (TILDA) or EMR-extracted (CPCR cohort). We also included the following baseline covariates: age group (70–74 years, 75–79 years, or ≥80 years) gender, marital status (married, separated/divorced, widowed, or never married/single), living arrangements (husband/wife/partner, family/relatives, living alone, or other), education (basic, or secondary/higher), social class (skilled, or unskilled), private health insurance and number of regular medicines (0–4, 5–9, or ≥10 medicines).

### Statistical analysis

Descriptive statistics of the CPCR and TILDA cohorts are presented, and differences in baseline characteristics between participants in the two cohort participants were examined using significance tests (chi-square test for categorical variables and Mann-Whitney U test for non-normal continuous variables). Descriptive statistics of healthcare utilisation (rates of ED visits, GP visits and OPD visits and proportion of participants with an ED visit, are presented across the two cohorts.

To examine differences in healthcare utilisation associated with study participation (i.e. TILDA versus CPCR cohort), regression models were fitted for each outcome including a variable to indicate which study participants were in. Then multivariate models were fitted which adjusted for other participant characteristics. For rates of GP, OPD and ED visits, negative binomial regression was performed and results are presented as incidence rate ratios (IRR) with 95% confidence intervals (CI). For the outcome of any ED visit, generalised linear models were fitted using the Poisson distribution with robust standard error estimates, yielding risk ratios (RR) with 95% CI. Lastly, interaction terms were generated to test whether the magnitude of difference in healthcare utilisation between the two cohorts differed by participant characteristics. Interaction terms between cohort status and each of the covariates were added sequentially to the models. Interactions were retained if they provided a statistically significant improvement to model fit (p < 0.05) assessed using the likelihood ratio test.

## Results

### Study participants

In the CPCR cohort, a total of 372 participants (46.2%) were male. (See Table 1) The median age was 76 years (IQR 72–80 years) and the majority were classified as skilled in terms of social class (n = 609, 75.6%). In terms of living arrangements, 359 (44.5%) lived with a spouse/partner and 308 (38.2%) lived alone. A total of 391 participants (48.5%) were prescribed 5–9 regular medications, with 103 (12.8%) prescribed 10 or more. The median number of prescribed medications was 6 (IQR 3–8).

**Table 1:** Baseline descriptive statistics for participants in TILDA and the CPCR cohorts.

	TILDA (n = 1377)	CPCR cohort (n = 806)	
	Median (IQR)	Median (IQR)	p value
Age (years)	76 (72–80)	76 (72–80)	0.548
Number of regular medicines	4 (2–6)	6 (3–8)	<0.001
	**n (%)**	**n (%)**	
Age group			
70–75 years	591 (42.9)	318 (39.5)	0.249
75–79 years	433 (31.5)	276 (34.2)	
> = 80 years	353 (25.6)	212 (26.3)	
Gender			0.586
Male	619 (45.0)	372 (46.2)	
Female	758 (55.1)	434 (53.9)	
Marital status^a^			<0.001
Married	747 (54.3)	370 (45.9)	
Separated/Divorced	41 (3.0)	40 (5.0)	
Widowed	448 (32.5)	253 (31.4)	
Never married/single	141 (10.2)	142 (17.6)	
Living arrangements^a^			<0.001
Husband/Wife/Partner	653 (47.4)	359 (44.5)	
Family/Relatives	221 (16.1)	100 (12.4)	
Live alone	499 (36.2)	308 (38.2)	
Other	4 (0.3)	38 (4.7)	
Education^a^^,^^b^			<0.001
Basic education	645 (46.8)	493 (61.2)	
Upper and post-secondary	731 (53.1)	307 (38.1)	
Social class			0.001
Unskilled	255 (18.5)	197 (24.4)	
Skilled	1122 (81.5)	609 (75.6)	
Number of regular medicines			<0.001
0–4	800 (58.1)	312 (38.7)	
5–9	505 (36.7)	391 (48.5)	
> = 10	72 (5.2)	103 (12.8)	
Private health insurance^b^			0.123
No	711 (51.6)	444 (55.1)	
Yes	665 (48.3)	362 (44.9)	

^a^ In CPCR cohort, missing data on marital status missing and living arrangements for one participant (0.1%), and education for six participants (0.7%)

^b^ In TILDA cohort, missing data on education for one participant (0.07%), and health insurance for one participant (0.07%)

In the TILDA cohort, a total of 619 participants (45.0%) were male. (See Table 1) The median age was 76 years (IQR 72–80 years) and the majority were classified as skilled in terms of social class (n = 1122, 81.5%). Regarding living arrangements, a total of 653 (47.4%) lived with a spouse/partner and 499 (36.2%) lived alone. A total of 505 (36.7) were prescribed 5–9 regular medications and 72 (5.2) 10 or more. The median number of prescribed medications was 4 (IQR 2–6).

The study participants are described in Table 1. The two cohorts were similar with regards to mean age (76 years) and gender. There were some differences between the cohorts, a higher proportion of TILDA participants were married and had higher educational attainment compared to the CPCR cohort. TILDA participants were also taking less prescribed medicines (See Table 1).

### Outcome of interest: Healthcare utilisation

In the CPCR cohort, the annual rates of GP, OPD and ED visits based on EMR data were 6.30 (SD 4.63), 2.11 (SD 2.46) and 0.26 (SD 0.62) respectively, compared to 5.65 (SD 8.06), 2.09 (SD 5.83) and 0.32 (SD 0.84) based on self-report in the TILDA cohort. (See Table 2) The mean rate of GP practice nurse visits in the CPCR cohort was 1.55 (SD 2.25). The median annual number of GP and OPD visits in the CPCR cohort were 5 (IQR 3–8) and 1 (IQR 0–3) respectively compared to 4 (IQR 3–6) and 1 (0–2) in the TILDA cohort. In univariate regression analysis comparing healthcare utilisation between the two cohorts, TILDA study participants’ reported a lower frequency of GP visits (unadjusted IRR 0.90 (95% CI 0.84, 0.96), p = 0.002)), a greater frequency of ED visits (1.20 (0.98, 1.49), p = 0.083)), and no difference in OPD visits (unadjusted IRR 0.99 (95% CI 0.86, 1.13), p = 0.845)). (See Table 3)

**Table 2:** Healthcare utilisation in each cohort summarised by participant characteristics.

	Annual rate of GP visits		Annual rate of OPD visits		Annual rate of ED visits		Any ED visit	
	TILDA	CPCR cohort	TILDA	CPCR cohort	TILDA	CPCR cohort	TILDA	CPCR cohort
	Mean (SD)						n (%)	
Overall	5.65 (8.06)	6.30 (4.63)	2.09 (5.83)	2.11 (2.46)	0.32 (0.84)	0.26 (0.62)	290 (21.1)	155 (19.2)
Age								
70–75 years	5.22 (6.99)	5.84 (4.27)	2.29 (7.31)	1.90 (2.36)	0.27 (0.66)	0.23 (0.54)	119 (20.1)	56 (17.6)
76–80 years	5.72 (5.20)	6.80 (4.88)	2.00 (4.56)	1.99 (2.03)	0.36 (0.93)	0.27 (0.69)	96 (22.2)	49 (17.8)
>80 years	6.27 (11.73)	6.33 (4.77)	1.85 (4.17)	2.59 (3.00)	0.35 (0.97)	0.30 (0.62)	75 (21.2)	50 (23.6)
Gender								
Male	5.82 (9.39)	5.77 (4.45)	2.30 (4.80)	2.34 (2.61)	0.31 (0.75)	0.26 (0.61)	126 (20.4)	72 (19.4)
Female	5.50 (6.78)	6.75 (4.74)	1.91 (6.54)	1.92 (2.31)	0.32 (0.90)	0.26 (0.62)	164 (21.6)	83 (19.1)
Marital status^a^								
Married	5.03 (4.74)	5.86 (4.14)	2.17 (4.86)	2.07 (2.41)	0.30 (0.68)	0.22 (0.55)	159 (21.3)	64 (17.3)
Separated/Divorced	4.95 (4.12)	7.03 (5.47)	1.66 (3.17)	2.18 (2.24)	0.15 (0.42)	0.25 (0.87)	5 (12.2)	5 (12.5)
Widowed	6.64 (12.33)	6.88 (4.88)	2.10 (7.69)	2.06 (2.58)	0.34 (0.99)	0.30 (0.66)	91 (20.3)	50 (19.8)
Never married/single	5.96 (4.69)	6.16 (5.03)	1.70 (3.97)	2.31 (2.45)	0.40 (1.11)	0.32 (0.62)	35 (24.8)	36 (25.4)
Living arrangements^a^								
Husband/Wife/Partner	4.88 (4.14)	5.86 (4.09)	2.11 (4.83)	2.03 (2.41)	0.28 (0.65)	0.22 (0.55)	136 (20.9)	61 (17.0)
Family/Relatives	6.92 (14.42)	5.55 (3.91)	2.04 (4.05)	1.63 (2.31)	0.47 (1.16)	0.26 (0.58)	58 (26.2)	20 (20.0)
Live alone	6.08 (7.94)	7.07 (5.40)	2.08 (7.47)	2.32 (2.52)	0.29 (0.87)	0.32 (0.71)	94 (18.8)	65 (21.1)
Other	5.75 (4.03)	6.00 (3.53)	1.75 (2.87)	2.47 (2.65)	0.50 (0.58)	0.24 (0.43)	2 (50.0)	9 (23.7)
Education^a^^,^^b^								
Basic education	6.33 (10.85)	6.54 (4.74)	1.95 (4.77)	1.90 (2.19)	0.32 (0.86)	0.26 (0.61)	131 (20.3)	95 (19.3)
Upper & post-secondary	5.04 (4.22)	5.93 (4.45)	2.21 (6.62)	2.49 (2.82)	0.32 (0.82)	0.27 (0.63)	159 (21.8)	60 (19.5)
Social class								
Unskilled	5.98 (6.75)	6.53 (5.26)	1.42 (2.73)	1.86 (2.25)	0.31 (0.89)	0.33 (0.70)	47 (18.5)	45 (22.8)
Skilled	5.57 (8.33)	6.22 (4.41)	2.24 (6.31)	2.20 (2.52)	0.32 (0.83)	0.24 (0.59)	243 (21.7)	110 (18.1)
Number of regular medicines								
0–4	4.87 (4.57)	4.80 (3.51)	1.58 (4.03)	1.38 (1.72)	0.23 (0.63)	0.17 (0.45)	138 (17.3)	43 (13.8)
5–9	6.04 (7.51)	6.83 (4.73)	2.74 (7.95)	2.41 (2.51)	0.40 (1.02)	0.28 (0.65)	125 (24.8)	79 (20.2)
> = 10	11.49 (24.04)	8.80 (5.66)	3.15 (4.33)	3.21 (3.39)	0.65 (1.20)	0.48 (0.84)	27 (37.5)	33 (32.0)
Private health insurance^b^								
No	6.37 (10.44)	6.49 (4.95)	1.90 (4.52)	1.95 (2.30)	0.27 (0.62)	0.28 (0.64)	144 (20.3)	91 (20.5)
Yes	4.88 (4.14)	6.06 (4.21)	2.29 (6.96)	2.31 (2.63)	0.37 (1.02)	0.24 (0.59)	146 (22.0)	64 (17.7)

^a^ In CPCR cohort, missing data on marital status missing and living arrangements for one participant (0.1%), and education for six participants (0.7%)

^b^ In TILDA cohort, missing data on education for one participant (0.07%), and health insurance for one participant (0.07%)

**Table 3:** Difference in healthcare utilisation associated with TILDA participation versus CPCR cohort participation.

	Univariate regression		Multivariate regression ^a^	
	**Unadjusted IRR**	**p value**	**Adjusted IRR**	**p value**
**Annual rate of**				
GP visits	0.90 (0.84, 0.96)	0.002	0.99 (0.92, 1.06)	0.684
OPD visits	0.99 (0.86, 1.13)	0.845	1.09 (0.95, 1.25)	0.230
ED visits	1.20 (0.98, 1.49)	0.083	1.37 (1.10, 1.71)	0.005
	**Unadjusted RR**		**Adjusted RR**	
Any ED visit	1.10 (0.92, 1.31)	0.304	1.23 (1.02, 1.48)	0.028

^a^ Adjusted for age (continuous), gender, marital status, living arrangements, education, social class, number of regular medicines (continuous) and private health insurance

In multivariate negative binominal regression analysis, adjusted for relevant confounders of age, gender, number of prescribed medications, living arrangements, education, social class and private health insurance cover, there was no difference in reported numbers of GP visits (adjusted IRR 0.99 (95% CI 0.92, 1.06), p = 0.684)) or OPD visits (adjusted IRR 1.09 (0.95, 1.25), p = 0.230)) between the two cohorts. However, TILDA study participants reported 37% more ED visits (adjusted IRR 1.37 (1.10, 1.71), p = 0.005)) and considering the outcome of any ED visit, adjusted for the same confounders, TILDA participants were more likely to report any ED visit than CPCR cohort participants (adjusted RR 1.23 (95% CI 1.02, 1.48), p = 0.028)). (See Table 3)

Considering interactions, there was evidence of differences in GP visits between the two cohorts in subgroups based on living arrangements and private health insurance. Among those with private health insurance, TILDA participants reported significantly lower rates (S1 Fig) while among those living with family/relatives, TILDA participants reported significantly higher rates compared to CPCR cohort participants (S2 Fig). With respect to OPD visits, for each year increase in age, TILDA participants reported significantly lower rates, whereas for CPCR cohort participants’ rates were higher with increasing age (S3 Fig).

## Discussion

### Summary of findings

This study which compares two Irish prospective cohort studies involving older people demonstrates no difference between self-reported GP and OPD visit rates compared to manual extraction of these data from the GP EMR. However self-reported ED visits were 37% higher than manually extracted ED visits rates. This is likely due to ED visits being underreported in the GP EMR. While GPs should receive a hospital discharge summary if a patient is admitted to hospital, they may or may not receive notifications for ED visits and patients may not report these visits to their GP.

### Comparison with existing literature

The rates of GP and OPD visits reported in the current study are similar to recent Irish research examining healthcare utilisation rates, with average annual self-reported GP visits of 6.4 in those aged 65–74 years, rising to 8.4 visits in those aged ≥75 years.[17] In the same study annual OPD visits were reported as 2.4 in the 65-74-year age group and 1.8 in those aged ≥75 years.[17] In another Irish study (n = 6 general practices, purposively selected) that utilised manual extraction of data from the GP EHR, GP visits were higher in those aged over 70 years with an average of 8.56 visits annually compared to an average of 6.3 visits in the current study.[18] However, this study had a broader definition of GP visit than the current study, in that telephone GP consultations and out of hours GP visits were included, which may explain some of the discrepancy.

Over reporting of self-reported emergency hospital use has been highlighted in previous studies. In a recent Dutch prospective cohort study (aged ≥70 years, n = 790) comparing self-report and GP EMR healthcare utilisation rates, 8% of patients over reported emergency hospital admission compared to 5.2% who underreported this outcome.[19] A possible explanation for this was that the GP was often not informed about ED visits especially if the patient was admitted. This highlights the importance of contextual factors in determining healthcare utilisation rates, which will vary internationally depending on how such data are transferred between secondary and primary care. In a US prospective cohort study (aged ≥40 years with at least one symptomatic disease, n = 216) participants under reported physician visits but significantly over reported ED visits.[12] However, other prospective cohort studies have reported high concordance for emergency hospital use with lower concordance for GP visits and OPD use for self-reported healthcare utilisation by older people.[7, 11]

Increasing age is an important factor; in the current study as age increased, patients were more likely to report lower healthcare utilisation rates. Therefore, self-reported healthcare utilisation may be less reliable in the very old. Older age has also been linked to discordance in reporting of prescribed medicines. A study comparing TILDA-reported medication to administrative pharmacy claims found good agreement overall for most medication classes, but older participants underreported medications for bone diseases and analgesics.[19] Other factors such as education level and morbidity burden may impact upon self-reported healthcare utilisation rates, with previous studies indicating that younger, healthier individuals with higher education are more likely to self-report healthcare utilisation accurately.[3, 5]

### Strengths and limitations

Some previous studies have been able to compare self-reported and EMR or administrative databases healthcare utilisation rates within the same study population.[3, 20] This was not possible in this study but the two study populations were similar and recorded comparable socio-demographic and utilisation variables. However, there were some differences between the cohorts in that a higher proportion of TILDA participants were married, had higher educational attainment, and were less likely to be unskilled compared to the CPCR cohort. TILDA participants were also taking fewer prescribed medicines and were nationally representative, while the CPCR cohort participants were recruited from one geographical area in Ireland only.

There are few recent studies examining how healthcare utilisation measurement may impact upon the accuracy of reported rates, especially in older people. Most existing studies have relatively small sample sizes, are more than 10 years old and were conducted in North America.[9, 12, 21, 22] The current study has limitations. First, the CPCR cohort excluded patients receiving palliative care at baseline whereas this was not an exclusion criterion in the TILDA cohort. Both cohorts’ excluded patients with significant cognitive impairment at baseline and included community-dwelling older people only. Second, in the CPCR cohort GP visits were defined as face to face GP consultations during routine hours and thus excluded telephone consultations and out of hours GP visits. GP practice nurse visits were recorded separately. In TILDA, participants were asked the following question as part of an interview with a trained assessor ‘In the last 12 months, about how often did you visit your GP?’. As these definitions of a GP visit are not identical this may have impacted upon reported GP visit rates.

Participants in both cohorts had eligibility to the GMS scheme, for which the vast majority of people aged over 70 years have coverage. Although free access to healthcare has been shown to be an important determinant of GP visits among older people, its role in the comparison of utilisation rates could not be examined here.[23] From a methodological perspective, losses to follow up in both cohorts are low with clear reasons presented for those lost to follow up.

### Research implications

This study indicates that GP visits and OPD visits self-reported by older patients were similar to utilisation rates reported in patients’ GP EMR. In terms of generalisability, health care systems internationally vary in terms of their use of the EMR and how well primary and secondary care EMR systems are linked. These local and contextual factors will impact upon choice of healthcare utilisation measurement modalities in future studies, which may differ depending on the outcome of interest (e.g. GP visit vs. ED visit). It is important to clearly define each individual aspect of healthcare utilisation to allow comparability across studies.

The choice of self-report healthcare utilisation measurement approach may also influence reporting rates. In this study self-reported health care use was ascertained at interview which is different to other self-report measurement methods such as postal questionnaires, which tend to have lower response rates with potential selection bias in respondents.[24]

## Conclusions

This study indicates that self-reported rates of GP and OPD visits were similar but there were significantly higher rates of self-reported ED visits in comparison to the EMR recorded visits. However, this is likely to be due to ED visits being underreported in the GP EMR as GPs may not receive electronic notifications for ED visits and patients may not report these visits to their GP. It is important that contextual issues such as transfer of healthcare utilisation data between sectors, which are likely to vary in different healthcare systems and need to be accounted for in ascertaining healthcare utilisation rates.

## Supporting information

S1 Fig Differences between TILDA and CPCR participants GP visits according to private health insurance status. (DOCX)

S2 Fig Differences between TILDA and CPCR participants’ GP visits according to living arrangements. (DOCX)

S3 Fig Differences between TILDA and CPCR participants’ OPD visits according to age. (DOCX)

S1 File Research Ethics Committee letter regarding data sharing. (DOCX)
