# Supplementary material for: Self-report versus electronic medical record recorded healthcare utilisation in older community-dwelling adults: Comparison of two prospective cohort studies
Source: PLoS One. 2018 Oct 26;13(10):e0206201. doi: 10.1371/journal.pone.0206201 (PMC6203362; doi:10.1371/journal.pone.0206201)

**S2 Figure: Differences between TILDA and CPCR participants’ GP visits according to living arrangements**


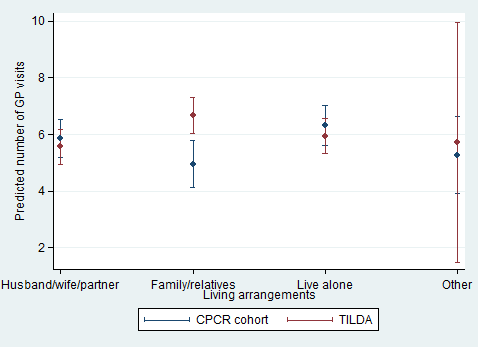

Supplement: S2 Fig — (DOCX) [file pone.0206201.s002.docx]
