# Supplementary figures and images for: Self-report versus electronic medical record recorded healthcare utilisation in older community-dwelling adults: Comparison of two prospective cohort studies
Source: PLoS One. 2018 Oct 26;13(10):e0206201. doi: 10.1371/journal.pone.0206201 (PMC6203362; doi:10.1371/journal.pone.0206201)

**S3 Figure: Differences between TILDA and CPCR participants’ OPD visits according to age**


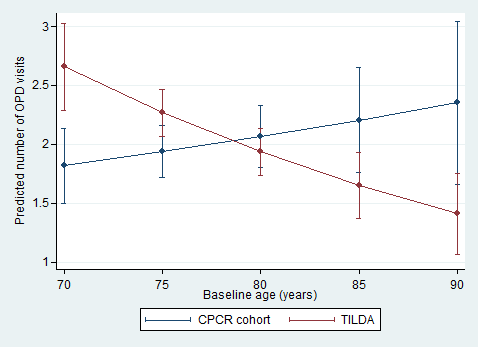

Supplement: S3 Fig — (DOCX) [file pone.0206201.s003.docx]

**S4 File**

**Letter from Research Ethics Committee regarding data sharing**


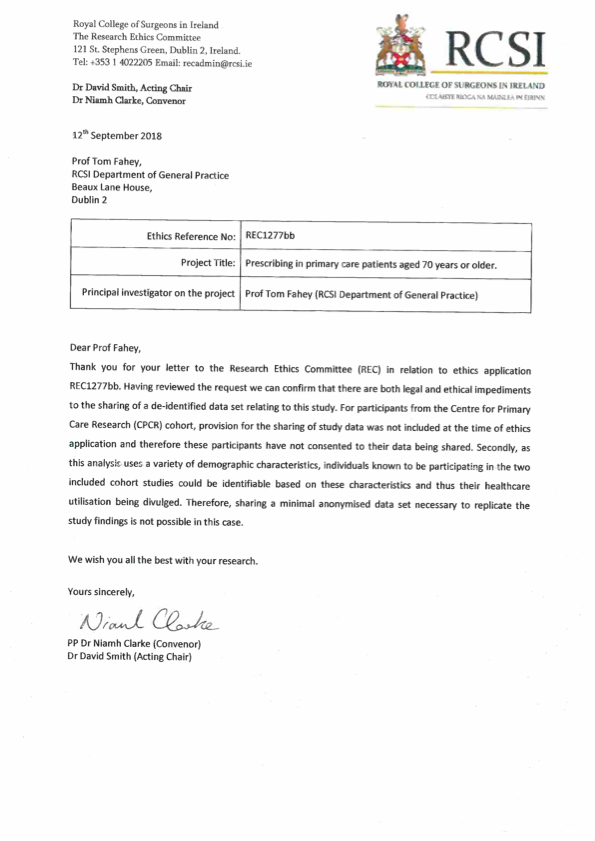

Supplement: S1 File — (DOCX) [file pone.0206201.s004.docx]
